# Analysis of the genetic diversity and population structure of *Salix psammophila* based on phenotypic traits and simple sequence repeat markers

**DOI:** 10.7717/peerj.6419

**Published:** 2019-02-18

**Authors:** Lei Hao, Guosheng Zhang, Dongye Lu, Jianjun Hu, Huixia Jia

**Affiliations:** 1 College of Forestry, Inner Mongolia Agricultural University, Hohhot, China; 2 Inner Mongolia University of Finance and Economics, Hohhot, China; 3 State Key Laboratory of Tree Genetics and Breeding, Key Laboratory of Tree Breeding and Cultivation of the State Forestry Administration, Research Institute of Forestry, Chinese Academy of Forestry, Beijing, China

**Keywords:** Genetic diversity, SSR, Structure genetics, Phenotypic traits, *Salix psammophila*

## Abstract

*Salix psammophila* (desert willow) is a shrub endemic to the Kubuqi Desert and the Mu Us Desert, China, that plays an important role in maintaining local ecosystems and can be used as a biomass feedstock for biofuels and bioenergy. However, the lack of information on phenotypic traits and molecular markers for this species limits the study of genetic diversity and population structure. In this study, nine phenotypic traits were analyzed to assess the morphological diversity and variation. The mean coefficient of variation of 17 populations ranged from 18.35% (branch angle (BA)) to 38.52% (leaf area (LA)). Unweighted pair-group method with arithmetic mean analysis of nine phenotypic traits of *S. psammophila* showed the same results, with the 17 populations clustering into five groups. We selected 491 genets of the 17 populations to analyze genetic diversity and population structure based on simple sequence repeat (SSR) markers. Analysis of molecular variance (AMOVA) revealed that most of the genetic variance (95%) was within populations, whereas only a small portion (5%) was among populations. Moreover, using the animal model with SSR-based relatedness estimated of *S. psammophila*, we found relatively moderate heritability values for phenotypic traits, suggesting that most of trait variation were caused by environmental or developmental variation. Principal coordinate and phylogenetic analyses based on SSR data revealed that populations P1, P2, P9, P16, and P17 were separated from the others. The results showed that the marginal populations located in the northeastern and southwestern had lower genetic diversity, which may be related to the direction of wind. These results provide a theoretical basis for germplasm management and genetic improvement of desert willow.

## Introduction

*Salix psammophila* (desert willow) is a shrub mainly distributed in Northwest China and is endemic to the Kubuqi Desert and the Mu Us Desert. *S. psammophila* (*Salix*; Salicaceae) is dioecious catkin-bearing and perennial shrub. It is about three to four m high. *S. psammophila* exhibits extraordinary adaptation to drought (Xiao & Zhou, 2001), high temperatures, wind erosion (Gao et al., 2004; Wang et al., 2006), sand burying, and abiotic stress (Liu et al., 2014a, 2014b). It is therefore planted to prevent wind erosion and control desertification, and is regarded as vegetation rehabilitation and forestation plant (Liu et al., 2015; Gao et al., 2006) that plays an important role in maintaining local ecosystems. With the gradual depletion of coal, oil, natural gas, and other non-renewable resources along with environmental degradation, the use of plants as renewable biomass resources and for reducing soil erosion has become an important topic. *S. psammophila* is easy to renew and grows rapidly, and is a promising biomass feedstock for biofuels and bioenergy (Guo et al., 2014; Li et al., 2013). It can also serve as a raw material for wood profiles, strengthening composite boards, and producing paper and activated carbon (Bao & Zhang, 2012; Li, Yang & Chen, 2012; Qu et al., 2012).

The phenotypic traits of *S. psammophila* are highly variable; individuals differ in terms of leaf size, plant height (PH), and branch size. Plant leaves utilize light energy for carbon fixation; branching pattern is the main factor determining plant architecture, and is also an important trait for the use of plants as raw materials in the above-mentioned applications. Moreover, *S. psammophila* is a dioecious shrub, females and males may perform differently to ecological stress (Wheeler et al., 2016). As such, investigating the phenotypic diversity of different populations and trait variation of sex dimorphism in *S. psammophila* could yield useful information for the genetic improvement of this species.

Molecular markers such as restriction fragment length polymorphisms (Brunner et al., 2001; Thormann, Myrholm & Mallett, 2001; Tsarouhas, Gullberg & Lagercrantz, 2002), amplified fragment length polymorphisms (Chen et al., 2016; Hu, Lv & Lu, 2011), simple sequence repeats (SSRs) (He et al., 2015; Perdereau et al., 2014; Cortés et al., 2014) and single nucleotide polymorphisms (Kim et al., 2018) have been widely used in *Salix* genome analyses. SSRs have a high rate of polymorphism, good reproducibility, and codominant inheritance, and are evenly distributed in coding and non-coding regions of the plant genome, making them ideal markers for studying genetic diversity and population structure (Kalia et al., 2010). Some polymorphic SSR markers have been developed and successfully used to analysis the genetic diversity and population structure in many willow species (Fogelqvist et al., 2015), such as *S. burjatica* (Barker et al., 2010), *S. viminalis* (Hanley et al., 2002), *S. arbutifolia* (Nagamitsu et al., 2014), *S. humboldtiana* (Bozzi et al., 2015), *S. herbacea* (Cortés et al., 2014) and so on. High levels of gene flow and genetic diversity of *S. caprea* have been inferred from chloroplast and nuclear SSR (Perdereau et al., 2014). Recently, compared with SSRs based on polyacrylamide gel electrophoresis, capillary electrophoresis had higher resolution (up to one bp) (Butler et al., 2004), and peak height ratios can be more easily calculated, allowing for allele ration determination when analyzing polyploid plant species (Palop, Segarra & Gonzalez, 2011). Jia et al. (2016) reported that some polymorphic SSR markers of *S. psammophila* have been developed by de novo transcriptome analysis. *S. psammophila* had been determined as naturally tetraploid based on chromosome counts, flow cytometry, and SSR analysis. Thus, SSR was ideal markers for studying genetic diversity and population structure of *S. psammophila*.

Animal-model analyses is a powerful approach to assess proportion of the additive genetic contribution to phenotypic trait variation and estimate heritability for the phenotypic traits (Klápště, Lstibůrek & El-Kassaby, 2014; Wilson et al., 2006). Animal model are linear mixed models that are based on pedigree or marker-inferred pairwise relatedness between individuals, and quantitative trait values of these individuals. It is widely used in wild animals (Husby, Visser & Kruuk, 2011; Réale et al., 2003), but rarely used in plants now. Sedlacek et al. (2016) used an animal model with SSR-based relatedness to estimate in natural populations of *S. herbacea*.

Therefore, we determined the variance of phenotypic traits and genetic diversity based on SSR, and tried to compare and analyze the genetic diversity and population structure of *S. psammophila* based on phenotypic traits and SSR. To this end, the present study analyzed nine quantitative phenotypic traits in 491 genets of 17 *S. psammophila* populations to evaluate morphological variation and clustering of phenotypic traits. Generalized linear mixed models were used to examine the effect of sex on phenotypic traits. Animal-model analysis was used to assess the heritability of phenotypic traits. Moreover, 22 SSR markers were used to assess genetic diversity, neighbor-joining (NJ) phylogenetic analyses, principal coordinate analysis (PCoA) and population structure of *S. psammophila*. Further, those results would provide a basis for germplasm resource management as well as breeding programs.

## Materials and Methods

### Plant materials

Research materials were collected from the germplasm resource preservation library of *S. psammophila* established in Ordos Dalad, Inner Mongolia in 2008. More than 1,000 genets were collected 21 populations from different areas. *S. psammophila* genets were randomly sampled within a 100 × 100 m^2^ plot at each population. The distance between each genets were at least 50 m. About 50 genets were collected from each population by phenotypic traits. We collected genets by phenotypic traits, including PH, ground diameter (GD), tree shape, branch color, and so on. According to phenotypic traits investigation, 3-year-old stem of branches with large and strong shrubs, no diseases and pests genets were selected to collect in the germplasm resource preservation library of *S. psammophila*.

**Figure 1:**
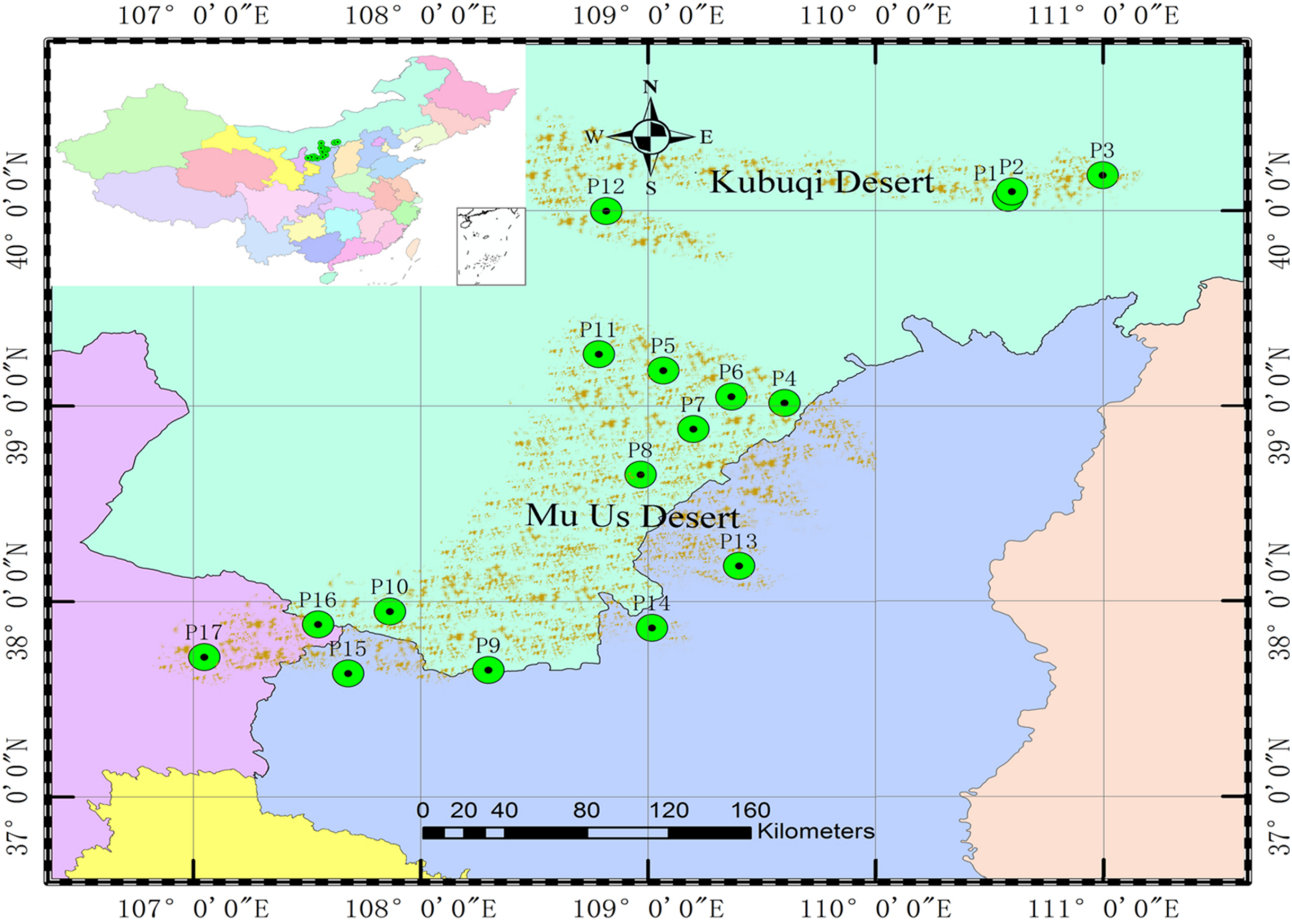
Locations of the 17 *S. psammophila* populations.

A total of 528 genets from 17 populations (P1–P17) were obtained from the germplasm collection to analyze population genetic structure and the association between SSR markers and specific phenotypic traits. There were 15 genets missing and recorded inaccurately in the phenotypic investigation, 22 genets of the same clones were detected by SSR markers; therefore, 491 genets were ultimately analyzed. The origins and locations of the 17 populations are shown in Table 1 and Fig. 1.

**Table 1:** Geographical characteristics of the 17 *S. psammophila* populations.

Location	Population	Number of clones	Latitude (N)	Longitude (E)	Elevation (m)
Wulanhao, Dalate, Inner Mongolia	P1	27	40°04′	110°35′	1,224
Baoshagedu, Dalate, Inner Mongolia	P2	22	40°06′	110°36′	1,128
Juhetan, Zhungeer, Inner Mongolia	P3	29	40°11′	111°00′	1,059
Hasake, Ejin Horo, Inner Mongolia	P4	32	39°01′	109°36′	1,125
Chahanzuoer, Wushen, Inner Mongolia	P5	28	39°11′	109°04′	1,081
Tuke, Wushen, Inner Mongolia	P6	29	39°03′	109°22′	1,156
Hulahu, Wushen, Inner Mongolia	P7	31	38°53′	109°12′	1,112
Aobao, Wushen, Inner Mongolia	P8	29	38°39′	108°58′	1,155
Chengchuan, Etuoke, Inner Mongolia	P9	29	37°39′	108°18′	1,194
Harise, Etuoke, Inner Mongolia	P10	32	37°57′	107°52′	1,187
Kaizhuoer, Etuoke, Inner Mongolia	P11	29	39°16′	108°47′	1,326
Wuritu, Hangjin, Inner Mongolia	P12	28	40°00′	108°49′	1,436
Qiaojiamao, Yulin, Shaanxi	P13	28	38°11′	109°24′	1,158
Majingou, Yulin, Shaanxi	P14	27	37°52′	109°01′	1,191
Dingbian, Yulin, Shaanxi	P15	32	37°38′	107°41′	1,362
Luoytuojing, Yanchi, Ningxia	P16	32	37°53′	107°33′	1,336
Haba Lake, Yanchi, Ningxia	P17	27	37°43′	107°03′	1,460

### Determination of phenotypic traits

Phenotypic traits of 22–32 genets in each population were evaluated in 2013, including nine quantitative phenotypic traits. Sex was recorded of each genets. PH and GD were measured with meter stick, and BA was measured with a goniometer (Fig. S1). Each genet was measured five different branches for BA. In addition, 8–13 leaves from each genet that grew well and had no signs of disease were photographed, and MapGIS 6.7 software (China University of Geosciences, Beijing, China) was used to measure and record the parameters of leaf length (LL), leaf width (LW), LA, leaf petiole (LP), and leaf perimeter (LPE). PH was measured as the distance from the ground level to the tip of the plant; GD was determined as the diameter of the plant at a height of 30 cm from the ground; BA was measured as shown in Fig. S1.

### DNA isolation and SSR analysis

Fresh and young leaves (0.2 g) were selected from each genet and total genomic DNA was isolated using a Plant Genomic DNA kit (TIANGEN, Beijing, China). The quality of DNA was verified by 1.2% agarose gel electrophoresis and a NanoDrop2000 spectrophotometer (Thermo Fisher Scientific, Waltham, MA, USA). The DNA was stored at −20 °C until used for PCR.

A total of 168 EST-SSR markers from the de novo transcriptome of *S. psammophila* including di- and tri-nucleotide repeats were randomly selected to design based on flanking sequences, and 125 pairs from 168 EST-SSR markers showed successful amplification (Jia et al., 2016). A total of 22 fluorophore-labeled SSR primers were selected (Table S1) and fluorophore-labeled M13 for TP-M13-SSR analysis (Schuelke, 2000). The 20 μl PCR reaction contained 100 ng template DNA, 10 × Taq buffer (Cat#ET101-02; TIANGEN, Beijing, China), 100 μM dNTP, 30 μM MgCl_2_, 0.5 unit Taq DNA polymerase (Lot#03330w; TIANGEN, Beijing, China), 2pmol forward primer, 8pmol reverse primer, 8pmol M13, and double-distilled H_2_O. The reactions were carried out in a 96-well ABI 9902 PCR instrument (Applied Biosystems, Foster City, CA, USA) under the following conditions: 94 °C for 5 min; 30 cycles of 30 s at 94 °C, 30 s at 57 °C, and 30 s at 72 °C; eight cycles of 30 s at 94 °C, 30 s at 57 °C, and 30 s at 72 °C; The last cycle was 72 °C for 10 min. PCR products was performed by capillary electrophoretic separation using an ABI 3730XL DNA Analyzer (Applied Biosystems, Foster City, CA, USA), and data were processed using Gene Marker v.2.2.0 software (Soft Genetics, State College, PA, USA).

### Statistical analysis

Differences in the nine quantitative phenotypic traits among 17 populations were evaluated by one-way analysis of variance using Data Processing System software (Tang & Zhang, 2012). Duncan’s multiple-range test was applied to compare the different populations. Quantitative traits were analyzed according to mean Euclidean distance based on genetic dissimilarity using DARwin 5 software (http://darwin.cirad.fr/), and the results were used to construct an UPGMA hierarchical clustering dendrogram of the 491 *S. psammophila* genets. Moreover, generalized linear mixed models were used to estimate the effect of sex on phenotypic traits (Wheeler et al., 2016). All statistical analyses were carried out in packages lme4 (Bates et al., 2014) and lmerTest (Kuznetsova, Brockhoff & Christensen, 2015) using R 3.2.3. Narrow-sense heritability (*h^2^*) of phenotypic traits were estimated with the multivariate restricted maximum likelihood (REML) animal model (Kruuk, 2004) using ASReml-R v.3.0 (Butler et al., 2007). The method of relatedness estimation using SSR genotypes and estimation of genetic parameters referred to study on *S. herbacea* (Sedlacek et al., 2016).

The microsatellite DNA allele counting-peak ratios method (Esselink, Nybom & Vosman, 2004) was used to read tetraploid genotypes based on calculated ratios between peak areas. All tetraploid genotypes were read twice according to marker motifs. Although reading peaks is a little complicated, estimating polyploid genotypes using SSRs can clearly differentiate within genus by high throughput genotyping with multiplexed PCR (Guo et al., 2006; Pyne et al., 2018), and is also an auxiliary tool for determining ploidy (Wu et al., 2018). Representative two-microsatellite loci are shown in Fig. S2. The weak peaks and smeared peak were excluded from the final data analysis. AUTOTET (Thrall & Young, 2000) was used to calculate allelic richness, allelic richness within individuals, observed heterozygosity (H_o_), expected heterozygosity (H_e_), and fixation coefficient (*F*). PIC_CALC v.0.6 (Nagy et al., 2012) was used to calculate the polymorphism information content (PIC) of genets. Genetic distances (Nei, Tajima & Tateno, 1983) between genets were calculated using Populations version 1.2.31 software (http://bioinformatics.org/populations/). The distance method was using Nei, Tajima & Tateno (1983). GenALEX6.5 (Peakall & Smouse, 2012) was used for PCoA using the genetic distance matrix obtained from the Populations v. 1.2.31 software. MEGA3.1 was used to construct a NJ phylogenetic tree of the 491 genets and 17 populations based on the genetic distance matrix. The genetic structure of the 491 genets was analyzed using STRUCTURE 2.3.4 software (Porras et al., 2013), which is based on the Bayesian model. The length of the burn-in period and the number of Markov Chain Monte Carlo replications after this period was assigned as 10,000 with an admixture. The structure was run 10 times at each *K* by setting *K* from 2 to 14.

## Results

### Phenotypic trait variation and heritability estimates

The phenotypic trait distribution of the 491 genets in 17 *S. psammophila* populations is shown in Table S2. P17 showed the highest values for leaf traits (LL, LA, LPE, LW, and LP). Average values were as follows: LL, 6.23 cm (range: 7.33 (P17)–5.27 (P8) cm); LPE, 13.95 cm (range: 16.54 (P17)–12.04 (P1) cm); PH, 212.2 cm (range: 258.59 (P9)–173.26 (P14) cm); GD, 12.50mm (range: 16.29 (P17)–5.28 (P13) mm); and BA, 31.29 (range: 37.62 (P1)–26.72 (P4)). The average coefficient of variation of nine phenotypic traits in different genets was 27.42%, ranging from 18.35% (BA) to 38.52% (LA) (Table 2). The results demonstrated that leaf traits have large variation. Comparing the average coefficient of variation of different populations, P1, P2, P11, P15, and P17 were found smallest coefficients of variation (21.16%, 20.32%, 17.41%, 20.96% and 19.72%, respectively). By contrast, P3, P4, and P5 populations had larger coefficients of variation (29.54%, 26.64%, and 29.25%, respectively), reflecting the richness of phenotypic variation. There was no significant difference in phenotypic traits between male and female genets (Table S3).

**Table 2:** Coefficients of variation of phenotypic traits in populations of *S. psammophila*.

Population	Trait									Mean
	LL	LA	LPE	LW	LL/LW	LP	BA	PH	GD	
P1	17.80	29.70	17.11	18.92	20.91	23.26	11.46	13.85	37.46	21.16
P2	16.72	29.83	16.56	17.50	18.49	24.00	11.30	11.87	36.57	20.32
P3	31.03	52.40	30.77	24.39	16.53	37.50	11.26	19.34	42.64	29.54
P4	24.49	36.41	24.73	18.92	26.91	40.00	16.28	14.09	37.92	26.64
P5	32.26	53.49	32.79	20.00	26.55	35.19	18.56	16.77	27.61	29.25
P6	20.86	38.42	20.46	25.00	19.56	25.49	12.46	14.54	30.20	23.00
P7	20.48	29.56	20.03	19.35	25.04	24.00	18.76	17.92	25.83	22.33
P8	20.87	28.86	20.56	20.00	27.34	25.53	9.24	15.21	28.32	21.77
P9	19.09	36.20	19.03	23.08	24.62	24.53	11.90	15.54	36.66	23.41
P10	23.04	33.33	23.29	16.13	24.44	33.96	15.22	16.66	24.06	23.35
P11	13.31	16.02	14.27	7.69	16.72	22.58	9.81	17.61	38.65	17.41
P12	21.04	30.39	20.80	17.65	32.05	34.00	11.43	15.85	29.80	23.67
P13	15.67	22.75	16.51	28.95	19.29	43.14	13.98	20.35	23.04	22.63
P14	17.92	31.90	22.22	22.86	22.80	25.00	14.95	19.11	20.27	21.89
P15	19.97	29.00	18.97	15.79	21.64	27.78	18.20	11.59	25.73	20.96
P16	18.27	26.79	32.80	16.86	16.48	25.00	8.22	23.93	33.38	22.41
P17	8.59	13.87	9.19	39.53	14.44	12.79	16.19	18.54	44.32	19.72
Total	23.46	38.52	24.41	25.23	24.73	33.71	18.35	20.12	38.24	27.42

Animal model revealed significant estimates of narrow-sense heritability (*h^2^*) for phenotypic traits, with the exception of LW and PH. For BA (*h^2^* = 0.255), LP (*h^2^* = 0.262) and GD (*h^2^* = 0.212), the *h^2^* estimates were relatively high. For LL (*h^2^* = 0.163), LA (*h^2^* = 0.136), LPE (*h^2^* = 0.163) and LL/LW (*h^2^* = 0.136), the *h^2^* estimates were relatively moderate (Table 3).

**Table 3:** Estimates of narrow-sense heritablity (h^2^) for phenotypic traits.

Traits	*h^2^* ± SE	lowCI	upCI	Va	Vr
LL	**0.163 ± 0.080**	0.007	0.319	**0.389**	**1.995**
LA	**0.136 ± 0.076**	−0.014	0.286	**0.087**	**0.554**
LPE	**0.163 ± 0.077**	0.012	0.314	**1.828**	**9.400**
LW	0.031 **±** 0.063	−0.093	0.154	0.000	0.006
LL/LW	**0.136 ± 0.076**	−0.012	0.284	**3.050**	**19.359**
BA	**0.255 ± 0.080**	0.099	0.412	**10.075**	**29.415**
LP	**0.262 ± 0.072**	0.120	0.404	**0.010**	**0.029**
PH	0.084 ± 0.071	−0.056	0.224	162.350	1,765.614
GD	**0.212 ± 0.074**	0.067	0.357	**6.719**	**24.978**

**Note:**

Significant values are in bold. lowCI and upCI was 95% confidence intervals; Va was the additive genetic variance; Vr was the residual variance.

### Clustering of phenotypic traits

**Figure 2:**
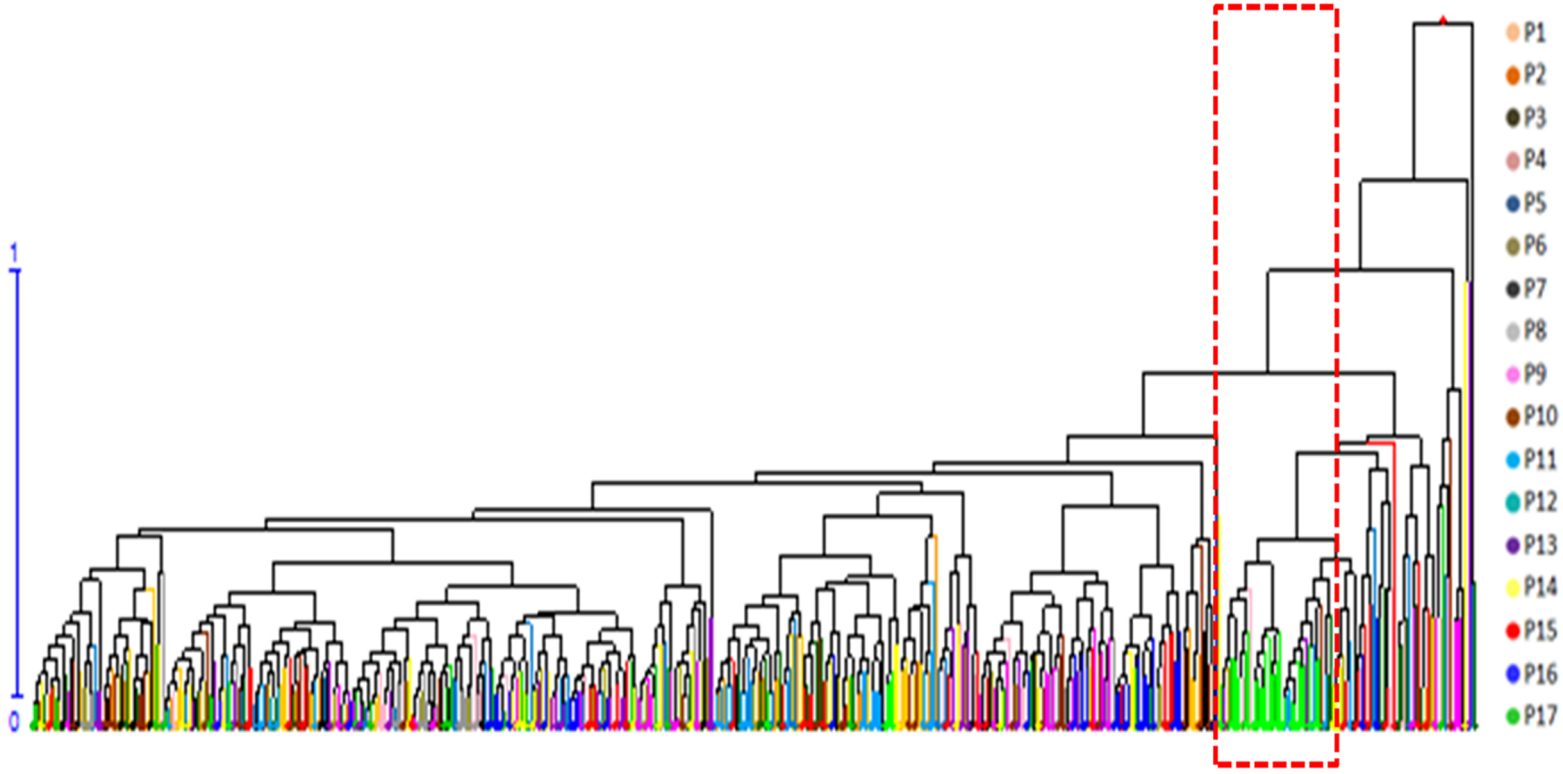
Hierarchical clustering dendrogram of 491 *S. psammophila* genets based on seven phenotypic traits.

A cluster analysis of seven phenotypic traits was carried out after removing PH and GD. PH and GD cannot be scored reliably because of stumping (3 years as a cycle) for shrubs, which are tufted. The seven phenotypic traits of the 491 genets were standardized and mean Euclidean distances obtained by the UPGMA method were used to construct a dendrogram (Fig. 2). The cluster analysis showed that genets from P17 were obviously isolated in the dendrogram, indicating that they possess unique characteristics.

**Figure 3:**
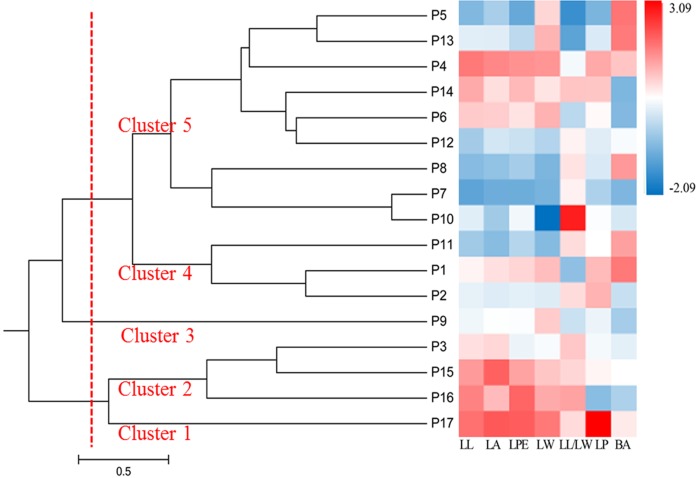
UPGMA dendrogram of 17 *S. psammophila* populations based on seven phenotypic traits. The gradual change of red to blue indicates that the average value of each phenotypic trait in the population is standardized range from 3.09 to −2.09.

Standardized average values of the seven phenotypic traits in different populations were used to generate a heat map of the 17 populations, which formed five clusters (Fig. 3). Cluster 1 was P17; Cluster 2 included P3, P15, and P16; Cluster 3 included P9, which separated into a single cluster; Cluster 4 consisted of P1, P2, and P11; and the remaining populations formed Cluster 5. The heat map showed that P15, P16, and P17 had the highest values for leaf phenotypic traits, whereas P1, P2, and P11 had the smallest. Populations in Clusters 1, 2, 3, and 4 were mostly from the southwestern and northeastern edges of the distribution area, suggesting that populations located in the periphery of the distribution area have phenotypic traits that are distinct from those of central populations.

### Genetic diversity analysis

A total of 22 SSR primers were used to analyze the 491 genets of 17 *S. psammophila* populations from Northern China. The mean sample size of each population was 27.85. The mean number of alleles per locus (Na) was 7.36 (range: 5.36–8.32). The average number of different alleles per individual and locus was 2.33 (range: 2.14–2.43). The mean genotypic richness (mean number of four-allele genotypes at a locus) was 14.5 (range: 6.68–18.00). Mean He (0.65) was higher than mean H_o_ (0.6), and mean PIC was 0.6 (Table 4). AMOVA revealed that most of genetic variance (95%) was within populations, with only a small portion (5%) occurring among populations (Table 5).

**Table 4:** Genetic variability statistics for 22 microsatellite loci in 17 *S. psammophila* populations.

Population	*N*	*A*	*Ai*	*G*	Ho	He	*F*	PIC
P1	26.36 ± 0.66	6.41 ± 2.92	2.37 ± 0.48	8.59 ± 3.43	0.62 ± 0.17	0.64 ± 0.15	0.03 ± 0.10	0.59 ± 0.16
P2	21.23 ± 1.41	5.36 ± 2.32	2.14 ± 0.54	6.96 ± 2.06	0.54 ± 0.21	0.59 ± 0.15	0.08 ± 0.13	0.54 ± 0.16
P3	28.59 ± 0.80	8.05 ± 3.26	2.43 ± 0.46	17.41 ± 6.52	0.62 ± 0.14	0.68 ± 0.12	0.09 ± 0.12	0.63 ± 0.14
P4	30.68 ± 1.62	7.82 ± 3.50	2.23 ± 0.36	17.46 ± 7.12	0.57 ± 0.12	0.65 ± 0.13	0.12 ± 0.09	0.60 ± 0.15
P5	26.27 ± 2.87	7.96 ± 3.37	2.30 ± 0.40	16.27 ± 6.09	0.59 ± 0.13	0.66 ± 0.12	0.10 ± 0.10	0.61 ± 0.14
P6	28.05 ± 0.84	8.05 ± 3.65	2.4 ± 0.43	16.32 ± 6.09	0.62 ± 0.14	0.67 ± 0.13	0.07 ± 0.11	0.62 ± 0.14
P7	29.64 ± 1.53	8.32 ± 3.46	2.27 ± 0.47	17.64 ± 6.99	0.58 ± 0.16	0.65 ± 0.15	0.11 ± 0.11	0.61 ± 0.16
P8	27.91 ± 1.77	7.50 ± 2.87	2.25 ± 0.41	16.05 ± 6.19	0.58 ± 0.14	0.65 ± 0.12	0.11 ± 0.10	0.60 ± 0.14
P9	28.46 ± 0.91	7.18 ± 2.97	2.33 ± 0.42	13.64 ± 4.48	0.60 ± 0.15	0.64 ± 0.13	0.07 ± 0.12	0.60 ± 0.14
P10	32.05 ± 1.53	7.59 ± 3.03	2.31 ± 0.38	18.00 ± 6.18	0.60 ± 0.13	0.66 ± 0.12	0.09 ± 0.08	0.61 ± 0.13
P11	28.41 ± 1.50	7.68 ± 3.15	2.39 ± 0.42	17.68 ± 6.68	0.62 ± 0.14	0.67 ± 0.13	0.07 ± 0.10	0.63 ± 0.15
P12	25.50 ± 2.45	7.64 ± 2.82	2.43 ± 0.43	14.68 ± 4.35	0.63 ± 0.14	0.67 ± 0.12	0.06 ± 0.08	0.63 ± 0.14
P13	26.77 ± 1.07	7.77 ± 3.61	2.45 ± 0.50	15.27 ± 5.33	0.63 ± 0.15	0.67 ± 0.12	0.07 ± 0.10	0.63 ± 0.14
P14	25.96 ± 1.36	7.27 ± 2.96	2.29 ± 0.45	13.46 ± 4.67	0.59 ± 0.15	0.64 ± 0.14	0.08 ± 0.09	0.60 ± 0.15
P15	30.86 ± 1.94	7.68 ± 3.36	2.31 ± 0.42	17.46 ± 7.27	0.59±0.13	0.64 ± 0.12	0.08 ± 0.07	0.60 ± 0.14
P16	30.05 ± 2.85	7.27 ± 3.06	2.24 ± 0.39	12.91 ± 4.17	0.57±0.14	0.63 ± 0.14	0.09 ± 0.10	0.59 ± 0.15
P17	26.64 ± 0.79	5.50 ± 2.37	2.4 ± 0.630	6.68 ± 3.03	0.62±0.21	0.57 ± 0.17	−0.09 ± 0.10	0.51 ± 0.17
Mean	27.85 ± 2.55	7.36 ± 0.84	2.33 ± 0.09	14.50 ± 3.75	0.60 ± 0.03	0.65 ± 0.03	0.07 ± 0.05	0.60 ± 0.03

**Note:**

*A*, mean number of alleles per locus; *Ai*, mean number of different alleles per individual and locus; *F*, fixation index; *G*, mean number of four allele genotypes at a locus; He, expected heterozygosity; Ho, observed heterozygosity; *N*, sample size; PIC, polymorphism information content.

**Table 5:** Analysis of molecular variance among and within *S. psammophila* populations.

Variation source	D*f*	Sum of squares	Estimated variance	Variation percentage (%)
Among populations	16	669.783	0.893	5
Within populations	474	7,623.771	16.084	95
Total variation	490	8,293.554	16.977	100

**Note:**

Df, degrees of freedom.

### NJ phylogenetic analysis, PCoA, and population structure

**Figure 4:**
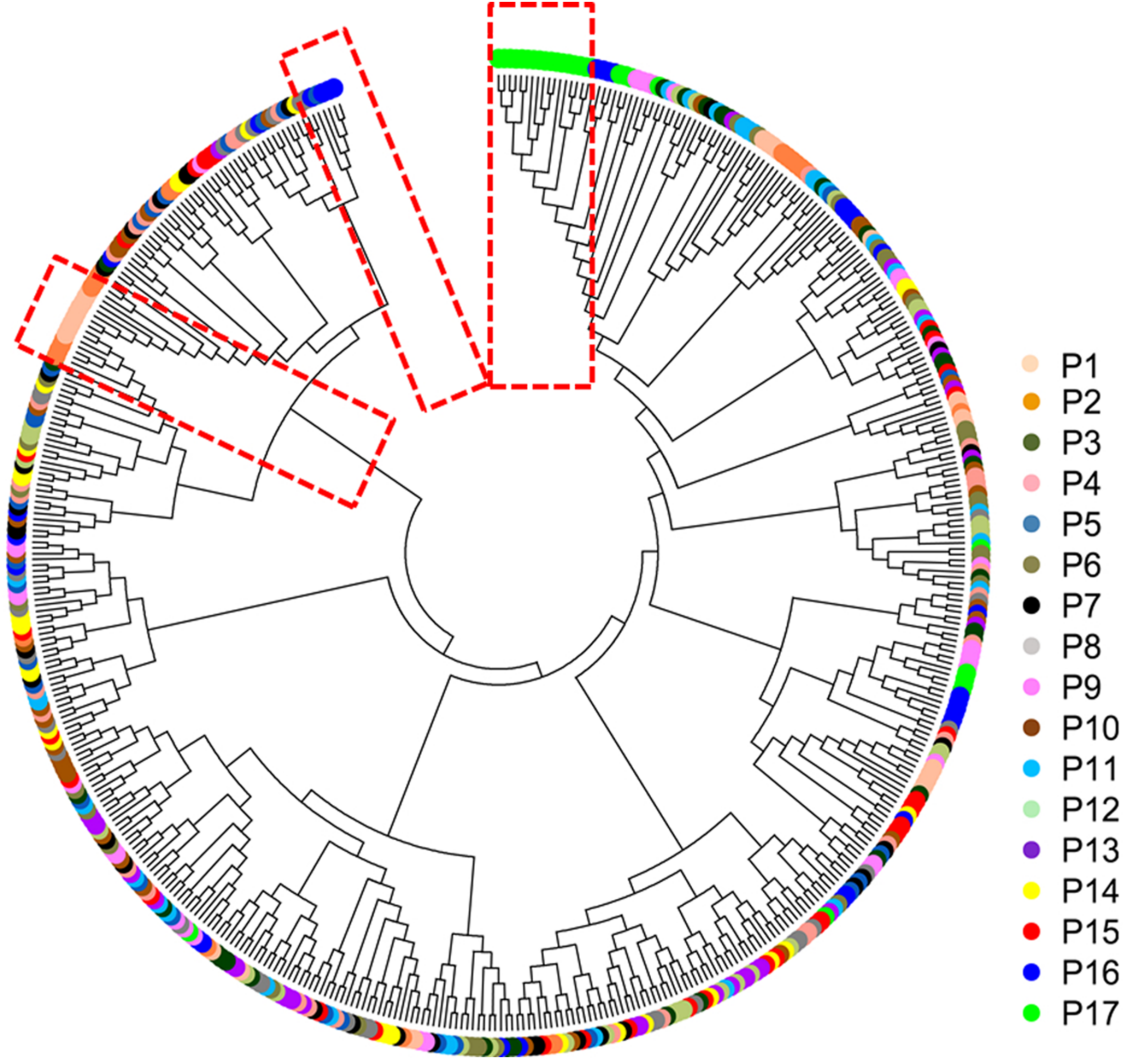
Neighbor-joining phylogenetic tree of 491 genets of *S. psammophila* based on SSR data.

**Figure 5:**
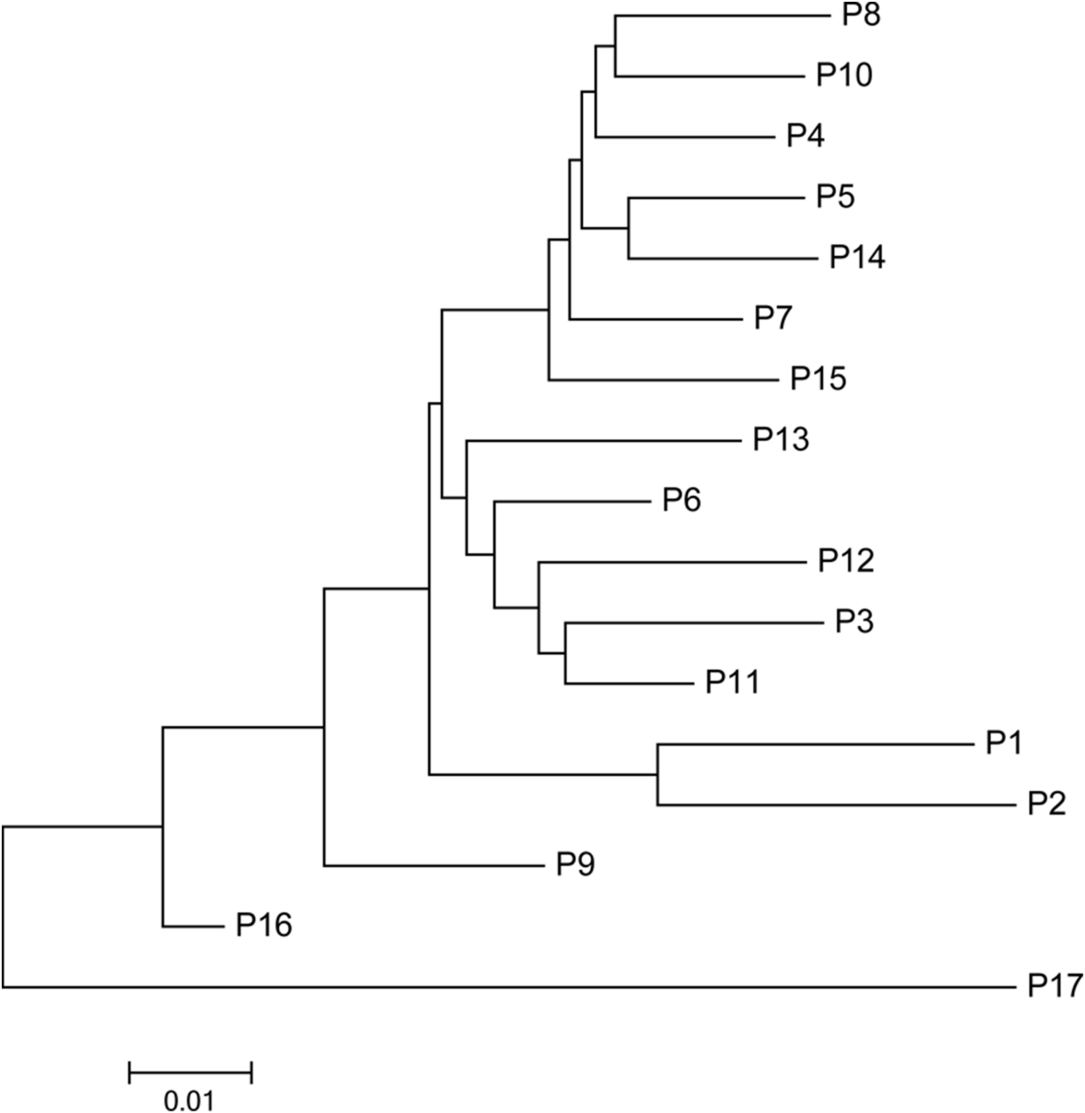
Neighbor-joining phylogenetic tree of 17 populations of *S. psammophila* based on SSR data.

The mean Nei’s genetic distance of the 491 genets of *S. psammophila* was 0.387 (range: 0.002–0.641). A NJ phylogenetic tree was constructed based on the calculated genetic distances (Fig. 4). The cluster analysis showed that individuals from P1, P2, P16, and P17 were distributed more centrally than other populations in the dendrogram, confirming the findings from the cluster analysis based on phenotypic traits. The population structure and NJ phylogenetic analyses indicated that P1 and P2 (from the northeastern part of the distribution area) and P9, P16, and P17 (from the southwestern part of the distribution area) were separated from other populations (Fig. 5). On the other hand, the results for P3, P15, and P11 based on SSR data were not consistent with the phenotypic trait analysis.

**Figure 6:**
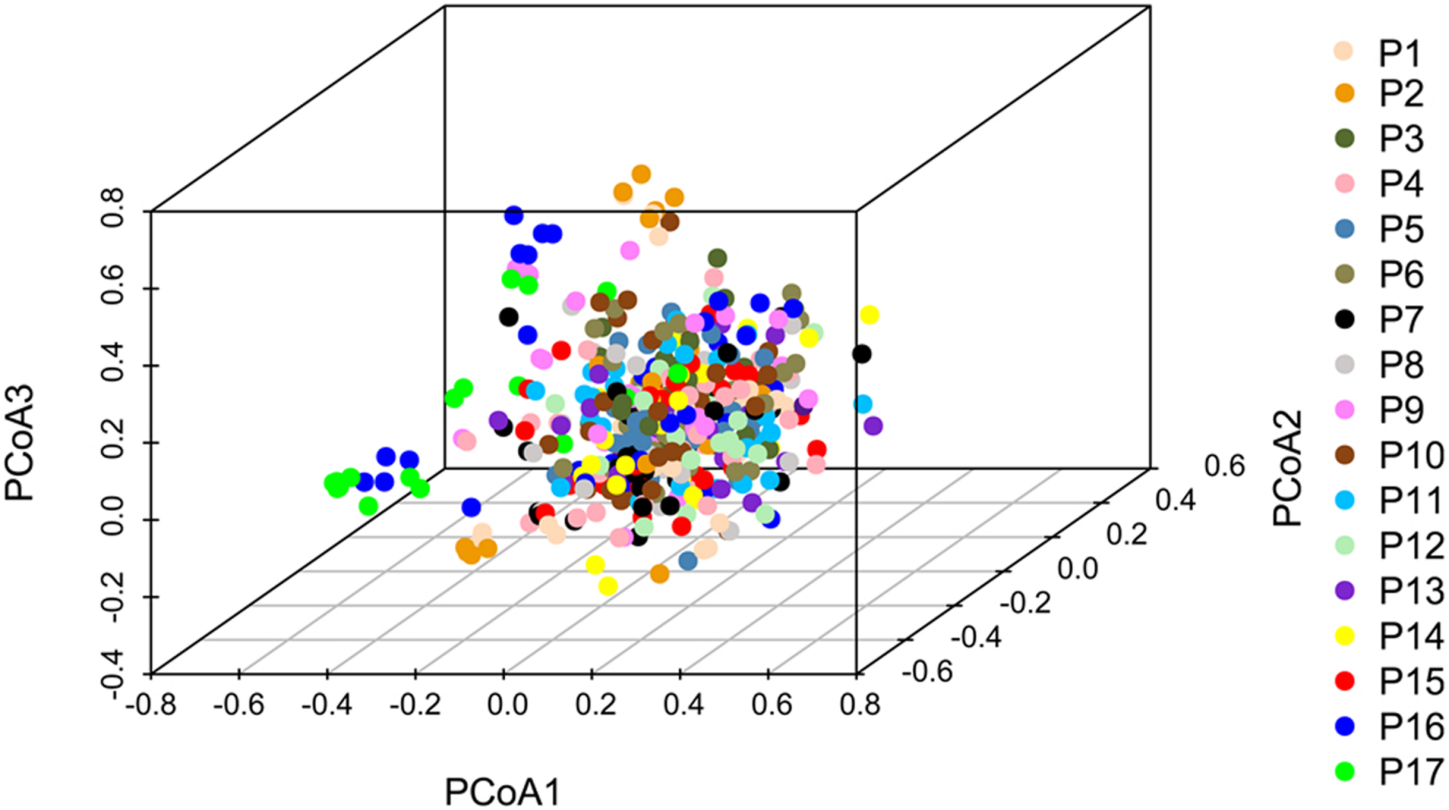
PCoA analyses based on SSR data for 491 genets from 17 *S. psammophila* populations. The 491 genets are labeled with different colors according to the population to which they belong.

Principal coordinate analysis based on SSR data revealed a large genetic diversity among the 491 genets. PCoA of first three axes explained 13.08% of the total variation (5.16%, 4.19%, and 3.74%, respectively), (Fig. 6). The clustering of genets in the PCoA was consistent with that in the NJ phylogenetic tree (Fig. 4), with P1, P2, P16, and P17 separated from the remaining populations. There were a lot of overlap genets of the center populations, and we inferred that those populations had more gene flow.

**Figure 7:**
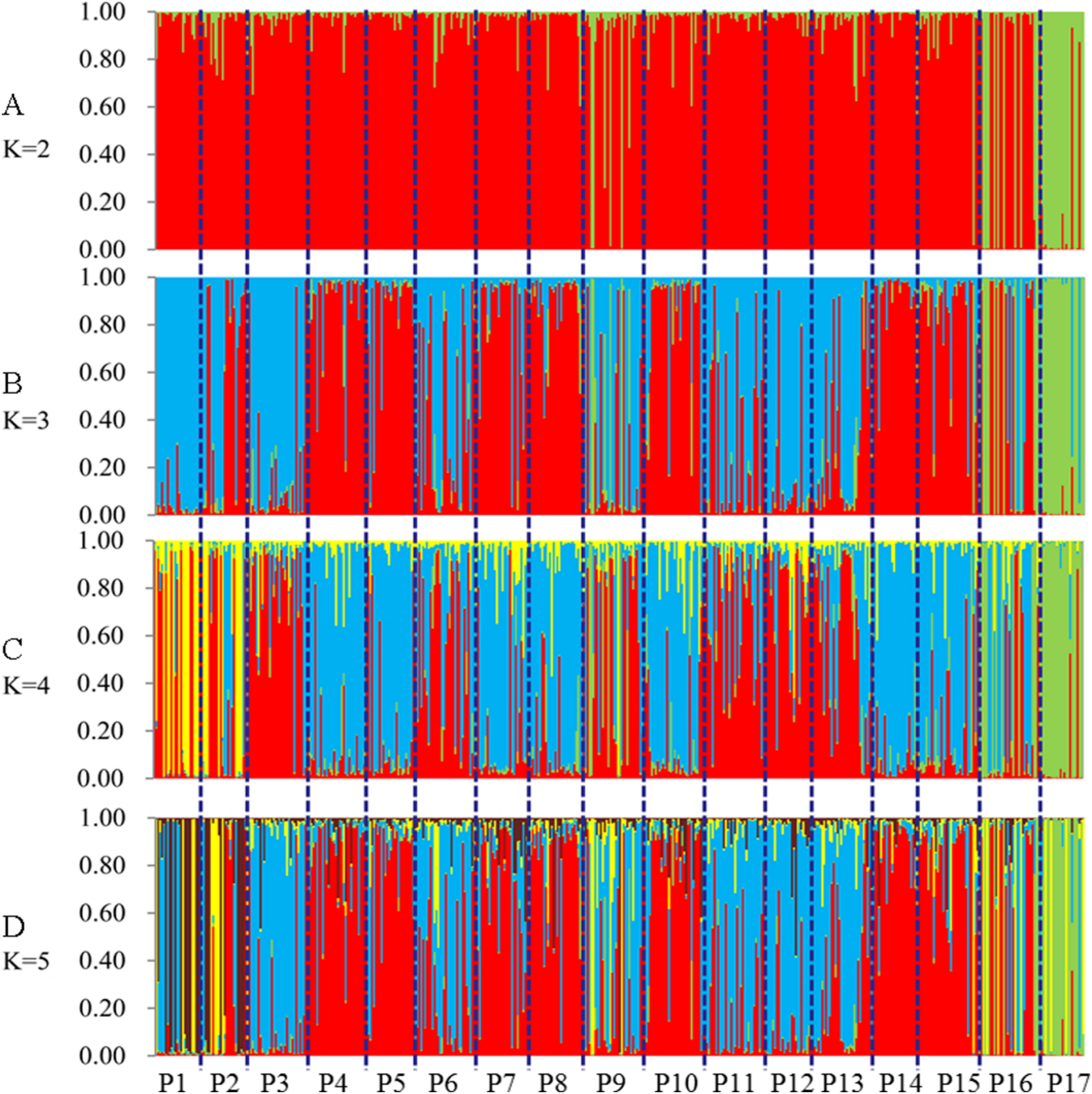
Population genetic structure of 491 genets from 17 populations of *S. psammophila* (2 ≤ *K* ≤ 5). (A) K = 2, (B) K = 3, (C) K = 4, (D) K = 5.

STRUCTURE software was used to analyze the genetic population structure of the 491 genets. *K* = 2 indicated that the populations could be clearly divided into two distinct subgroups. The highest proportion of genets assigned to the second population were P9 (21.2%), P16 (32.3%), and P17 (87.8%) at *K* = 2 (Fig. 7). P17 population always showed obvious particularity according to the analysis was run for 2 ≤ *K* ≤ 5.

## Discussion

There are about 330–500 species of *Salix* in the world. Many species show phenotypic plasticity (Karp et al., 2011). Phenotypic diversity is influenced by genetic and environmental factors (Korior et al., 2013). Willows are deciduous plants with simple, alternate leaves, and exhibit large variations in leaf size (Karp et al., 2011). For example, *S. viminalis* has long slender leaves, while *S. pentandra* has large broader leaves. In the present study, the mean coefficient of variation of the nine phenotypic traits in 17 populations ranged from 18.35% (BA) to 38.52% (LA). The results demonstrated remarkable variation in the leaves of *S. psammophila*. However, sex did not strongly affect responses in this dioecious species (Table S3). Using the animal model with SSR-based relatedness estimated 17 populations of *S. psammophila,* we found relatively moderate heritability values for phenotypic traits (Table 3). Estimating using animal model had lower heritability than other approaches (Postma, 2014). Gouker (2016) reported marker-based estimations of narrow sense heritability of all traits were calculated ranged from 0.29 to 0.87 based on genotypic means. In present study showed that the heritability values of phenotypic trait from BA (*h^2^* = 0.255) to LW (*h^2^* = 0.031), suggesting that most of the trait variation were caused by environmental or developmental variation. In the present study, the experimental materials were selected from the germplasm resource preservation library with same growth environment. This might be a reason why the heritability values were higher than for *S. herbacea* (Sedlacek et al., 2016). We suggested that the significant heritable variance in phenotypic traits might help *S. psammophila* to adapt to a changing environment. In other words, breeding values were also calculated based on heritability (Table S4). The largest five genets of breeding values were selected as useful germplasm material for phenotypic traits.

A high degree of genetic diversity was also observed in the 491 *S. psammophila* genets on the basis of analysis using 22 SSR markers: the Na was 7.36 and mean He was 0.65, which was comparable to the genetic diversity reported in *S. viminalis* (Na = 13.46, He = 0.616) (Berlin et al., 2014), *S. caprea* (Na = 10, He = 0.58) (Perdereau et al., 2014), *Populus nigra* (Na = 15.9, He = 0.47) (Jiang et al., 2015). *S. cheilophila* distributed in the Kubuqi Desert and Mu Us Desert, and often formed willow forests with *S. psammophila*. Therefore, *S. psammophila* frequently hybridizes and its tetraploidy might have contributed to its high degree of genetic diversity. The AMOVA indicated that most of the variation in *S. psammophila* (95%) occurred within populations. This is consistent with the analysis of SSR genetic diversity in *S. viminalis* (94%) (Zhai et al., 2016), and *P. nigra* (90.8%) (Jiang et al., 2015). The geographical distance between the two farthest populations (P3 and P17) is only 438 km; this would give rise to more gene flow between populations through pollen and seed dispersal. Thus, dioecious pollination in *S. psammophila* restricted to a comparatively small area could result in increased gene flow across generations and cause remarkable variations within populations (Hamrick & Godt, 1996).

Comparison of cluster analyses based on nine phenotypic traits as well as SSR data of *S. psammophila* populations showed that P1, P2, P9, P16, and P17 were distinct from the other populations. This was confirmed by analysis of population structure and PCoA analysis based on SSR data. Notably, all these separated populations were from the periphery of the distribution area—P1 and P2 from the northeast, P9 from the center, P16 and P17 from the southwest. The 17 populations of *S. psammophila* basically covered all of them in the Kubuqi Desert and Mu Us Desert, and the populations formed belt distribution from the northwest to the southeast. The Salicaceae species are dioecious, and exhibit a combination of anemophilous and entomophilous pollination (Peeters & Totland, 1999). *S. psammophila* mainly depends on wind pollination; its flowers usually bloom in early April and fruits ripen in early May. The seeds of *S. psammophila* are very small, with a 1,000-seed weight of approximately 0.08 g, which can facilitate dispersal by wind (Zhang et al., 2013). Wind direction, speed, and persistence play important roles in pollen transport, particularly when weak winds prevail for a considerable part of the year (Damialis et al., 2005). However, the northwest winds mainly occur in this region from April to May. This hinders genetic flow between P1, P2, P16, and P17 (marginal populations and upstream in wind direction) and other populations. In present study, we also found that these populations had relatively smaller coefficient of variation in phenotypic traits (21.16%, 20.32%, 22. 41%, and 19.72% in P1, P2, P16, and P17, respectively), and lower PIC based on SSR (0.59, 0.54, 0.59, and 0.51 in P1, P2, P16, and P17, respectively). Although P9 was also located on the marginal parts of the distribution area, this population was rich in *S. psammophila* in the upstream direction of the wind. Therefore, P9 had higher coefficient of variation in phenotypic traits (23.41%) and PIC (0.6) than P1, P2, P16, and P17. The results showed that the marginal populations located in upstream direction of wind had lower genetic diversity, which may be related to the pollination pattern of the wind. P3, P11, and P15 were clustered separately on the basis of distinct phenotypic traits but not on the basis of SSR data. This can be explained by the fact that the expression of plant traits is the result of the interaction between genotypes and the internal and external environments. Moreover, SSR markers are a simple motif that cannot cover the entire genome, whereas phenotypic traits are influenced by specific gene loci (He et al., 2015; Zhang et al., 2010).

In the cluster analysis of all individuals on the basis of phenotype (Fig. 2) and NJ phylogenetic analysis (Fig. 4) showed that the genets from the P17 population were obviously isolated from all individuals, showing more obvious particularity. First, we found that P17 population showed larger leaves in the phenotypic study. P1–P16 were located in the Kubuqi Desert and Mu Us Desert with typical desert habitats, while the P17 genets were sampled around Haba Lake with wetland habitats. Environmental heterogeneity in the original populations may induce adaptive genetic differentiation, which in turn results on phenotypic variability. The studied on populations of *S. herbacea* can respond to shifts in snowmelt by plastic changes in phenology and leaf size (Sedlacek et al., 2015). Potential drivers of phenotypic variability in *Salix*, such as community facilitation and competition (Wheeler et al., 2015), nutrient availability (Little et al., 2016), and abiotic stress (Wheeler et al., 2014), which response in phenotypic plasticity can help plant adapt to the new environment. Moreover, Sedlacek et al. (2014) indicated that soil inocula from different origins affected seed germination rates and growth of *S. herbacea* and interaction with soil biota may constrain migration of *S. herbacea* to higher altitudes. *S. psammophila* performs well in dry conditions and can be used to cope with soil erosion, plant-soil interactions also may play in the habitat suitability. Second, P17 was located in the southwestern part of the distribution area, with upstream wind direction, and at a relatively greater distance from the surrounding population P16 (approximately 47.733 km). Gene flow was mainly found to be affected by the distance between the donor and acceptor plants (Rognli, Nilsson & Nurminiemi, 2010). Therefore, these factors may lead to low gene flow with other populations. The present study showed that the genets from the P17 population had relatively smallest coefficient of variation in phenotypic traits (19.72%) and PIC (0.51), and therefore had lowest genetic diversity, showing more obvious particularity of characteristics.

## Conclusions

In the present study, we analyzed 491 genets from 17 natural populations of *S. psammophila* to evaluate genetic diversity, population structure based on morphology and SSR markers. Our results suggest that leaf variation of *S. psammophila* varies richly. The frequently hybridize and the tetraploidy of *S. psammophila* may have contributed to its high degree of genetic diversity. The results showed that the marginal populations located in the northeastern and southwestern had lower genetic diversity, which could be related to the direction of wind. Moreover, we found relatively moderate heritability values for phenotypic traits using the animal model with SSR-based relatedness estimated, suggesting that most of the trait variation were caused by environmental or developmental variation. Nonetheless, the findings presented here provide a theoretical basis for improving *S. psammophila* germplasm resources as well as useful information for breeding programs. Further research should advance in understanding the potential drivers of phenotypic variability and pay more attention to marginal populations of *S. psammophila*.

## Supplemental Information

10.7717/peerj.6419/supp-1 Supplemental Information 1 Determination of branch angle.

10.7717/peerj.6419/supp-2 Supplemental Information 2 Primers c-100 (green) and c-74 (blue) in electropherograms of five clones.

10.7717/peerj.6419/supp-3 Supplemental Information 3 PCR products amplified using 22 SSR primers in *S. psammophila.* A, mean number of alleles per locus; Ai, mean number of different alleles per individual and locus; F, fixation index; G, mean number of four allele genotypes at a locus; He, expected heterozygosity; Ho, observed heterozygosity; N, sample size; PIC, polymorphism information content; SSR, simple sequence repeat.

10.7717/peerj.6419/supp-4 Supplemental Information 4 Statistical parameters (mean ± standard deviation) of phenotypic traits in 17 *S. psammophila* populations. BA, branch angle; GD, ground diameter; LA, leaf area; LL, leaf length; LP, leaf petiole; LPE, leaf perimeter; LW, leaf width; PH, plant height. Means with different letters within a column are significantly different according to Duncan test (*P* < 0.01).

10.7717/peerj.6419/supp-5 Supplemental Information 5 Responses of phenotypic traits to sex of *S. psammophila*. The data are from general linear mixed models. numDF: numerator degrees of freedom. denDF: denominator degrees of freedom. Estimate, *t*- and *P*-values from the mixed model.

10.7717/peerj.6419/supp-6 Supplemental Information 6 Estimated breeding values and order for phenotypic traits of *S. psammophila*.

10.7717/peerj.6419/supp-7 Supplemental Information 7 Raw data.

## Additional Information and Declarations

### Data Availability

The following information was supplied regarding data availability: Raw data are available in a Supplemental File.
