# Supplementary figures and images for: Analysis of the genetic diversity and population structure of Salix psammophila based on phenotypic traits and simple sequence repeat markers
Source: PeerJ. 2019 Feb 18;7:e6419. doi: 10.7717/peerj.6419 (PMC6383557; doi:10.7717/peerj.6419)

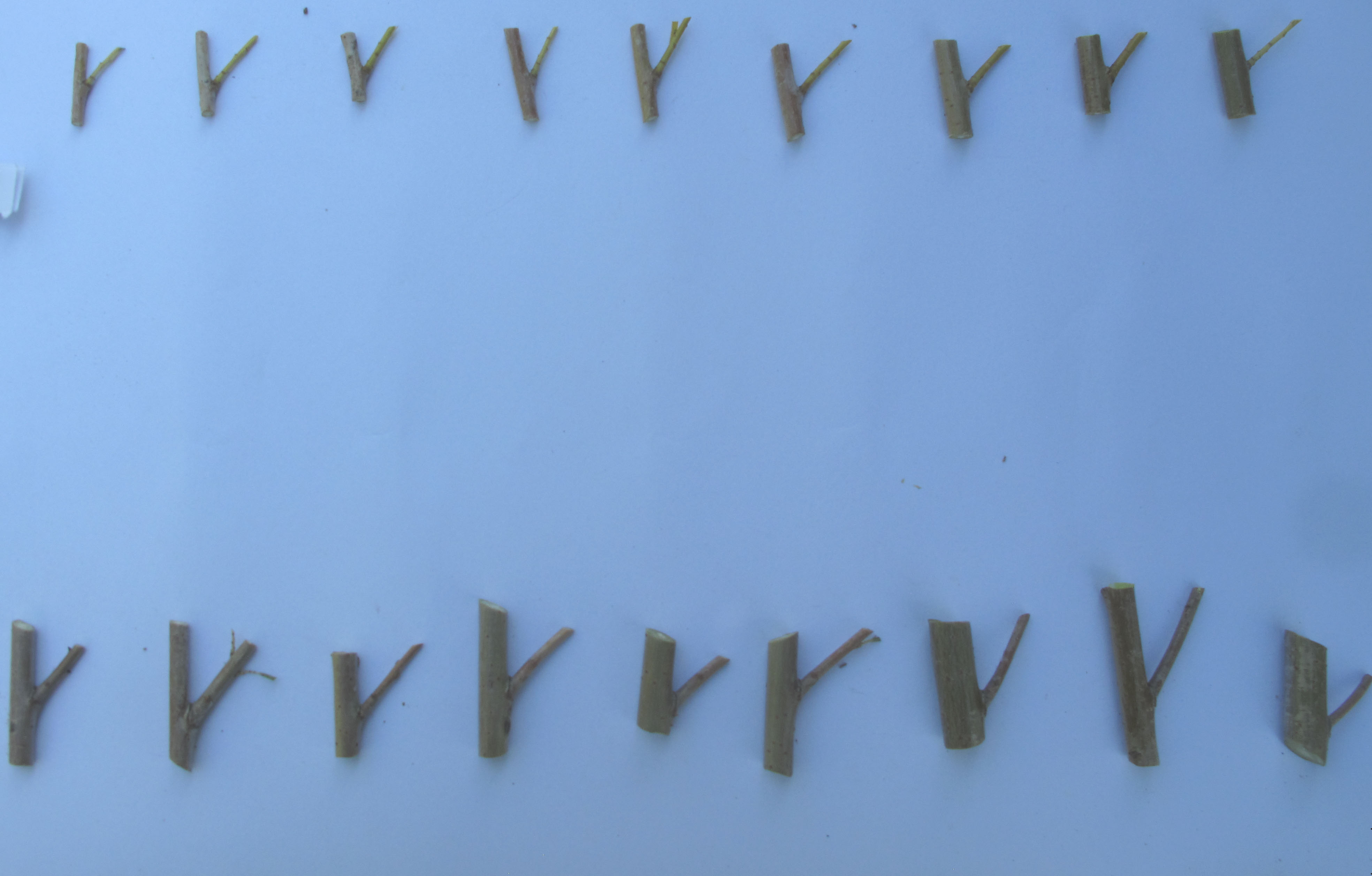

Supplement: Supplemental Information 1 [file peerj-07-6419-s001.jpg]

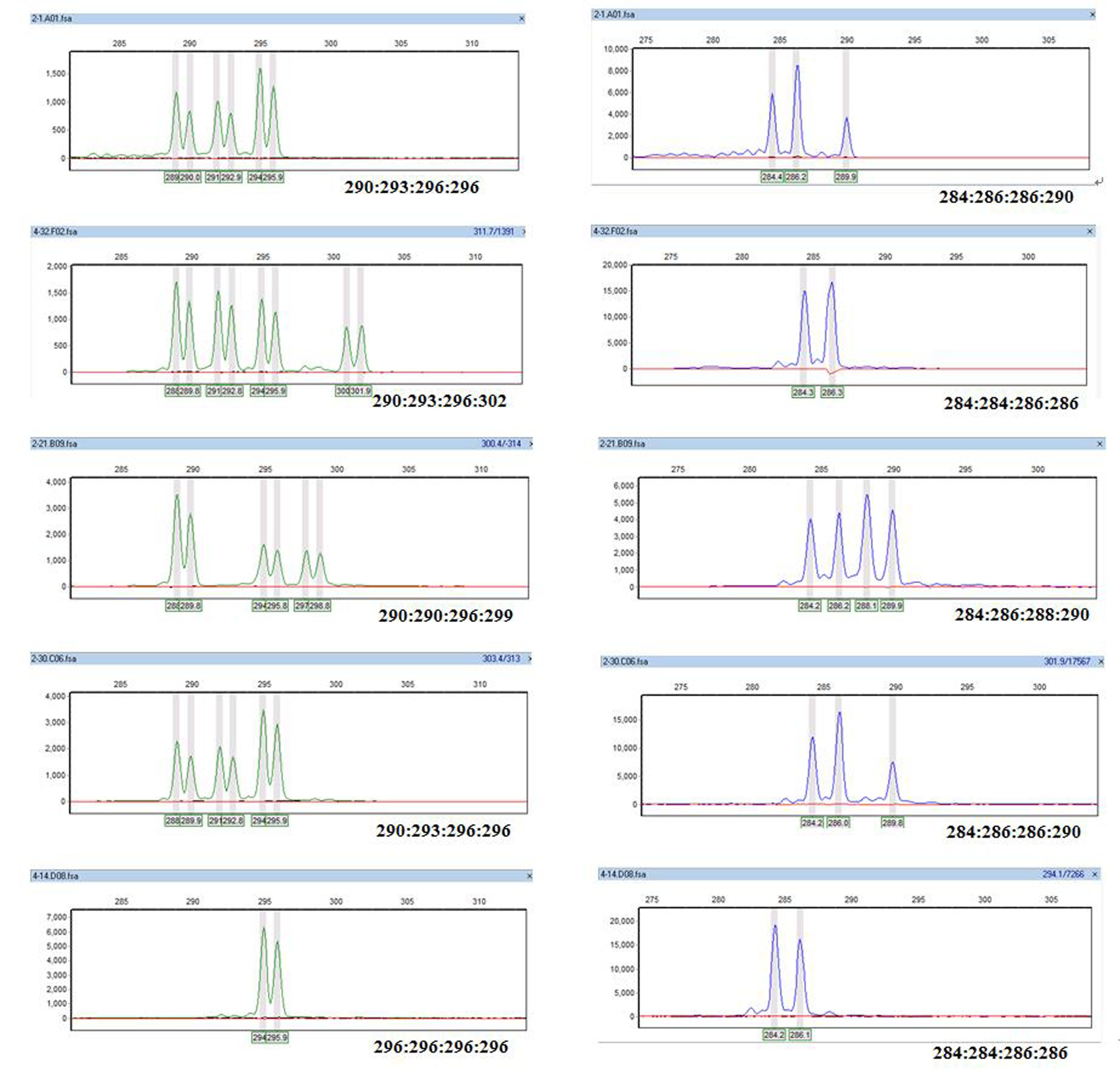

Supplement: Supplemental Information 2 [file peerj-07-6419-s002.jpg]
