# Supplementary material for: Analysis of the genetic diversity and population structure of Salix psammophila based on phenotypic traits and simple sequence repeat markers
Source: PeerJ. 2019 Feb 18;7:e6419. doi: 10.7717/peerj.6419 (PMC6383557; doi:10.7717/peerj.6419)
Supplement: Supplemental Information 3 — A, mean number of alleles per locus; Ai, mean number of different alleles per individual and locus; F, fixation index; G, mean number of four allele genotypes at a locus; He, expected heterozygosity; Ho, observed heterozygosity; N, sample size; PIC, polymorphism information content; SSR, simple sequence repeat. [file peerj-07-6419-s003.docx]

**Table S1.** PCR products amplified using 22 SSR primers in *S. psammophila*

| **Locus** | **Repeat motif** | **Primer sequence (5'→3')** |  | **Size (bp)** | **A** | **Ai** | **G** | **Ho** | **He** | **PIC** |
| --- | --- | --- | --- | --- | --- | --- | --- | --- | --- | --- |
| c4 | (AG)_8_ | F:CTTCCACATGCCTCTGACAA | R:TTGGACACAGACACGCTTTT | 240–266 | 12 | 2.428 | 114 | 0.611 | 0.664 | 0.641 |
| c16 | (TTC)_5_ | F:CTTCTCGGCTTCAACTTTCG | R:ACAATTCCAATAACCCGCAG | 211–238 | 8 | 2.196 | 33 | 0.549 | 0.550 | 0.504 |
| c24 | (GT)_8_ | F:ATGGAGATCAGCAGTGAGCC | R:TTGCTCTGGGGATTTTCTTG | 257–287 | 14 | 2.812 | 138 | 0.751 | 0.761 | 0.737 |
| c25 | (TG)_6_ | F:TTCACGTCCTCTCTTTGCCT | R:CCTCTAGAGTGCTTGCAGGG | 186–198 | 5 | 1.833 | 9 | 0.473 | 0.460 | 0.359 |
| c46 | (TCC)_7_ | F:TTCAAGCAAACGCCTTCTTT | R:TGAACAGTGGGACCAGATGA | 206–227 | 8 | 2.514 | 64 | 0.670 | 0.668 | 0.619 |
| c49 | (TGG)_5_ | F:GGAAGGGTTAGGGTTATGGG | R:TAAAACGGATACAGGGAGCG | 178–199 | 8 | 1.964 | 34 | 0.456 | 0.482 | 0.445 |
| c52 | (GA)_8_ | F:CGTTGTGTGGATTGTTTTCG | R:TGGTGGAATCACCACTTCAA | 216–260 | 17 | 2.146 | 131 | 0.525 | 0.621 | 0.606 |
| c57 | (TTC)_5_ | F:GCCCACCTACCTACAACGAA | R:TTTCTCCAGAGCTCCCTTCA | 205–214 | 4 | 1.773 | 11 | 0.414 | 0.428 | 0.351 |
| c59 | (TC)_7_ | F:TGATAGGTGCGCAGTTTTTG | R:TCCGTACTTGCCGGTTTATC | 236–272 | 19 | 3.320 | 274 | 0.874 | 0.877 | 0.867 |
| c61 | (GA)_9_ | F:GGGAGACTTGTGCGTTTGAT | R:AAAGCGTTCTGGTTTGGTAA | 232–264 | 16 | 2.779 | 190 | 0.750 | 0.838 | 0.822 |
| c69 | (GA)_8_ | F:CGAAGTTCTTAAAACCATCA | R:CCCACTCCATCTCTGGATTC | 237–269 | 16 | 2.701 | 238 | 0.731 | 0.884 | 0.873 |
| c73 | (AC)_6_ | F:TGAATTAGGGTTTCTCCCCC | R:AAAGCCTTCTGGGCTCTCTC | 328–344 | 9 | 2.261 | 104 | 0.563 | 0.750 | 0.715 |
| c74 | (GA)_7_ | F:ATTGCCAATTGTCAGCTCCT | R:AACCATGCCCACAAGAAAAG | 284–294 | 6 | 2.395 | 37 | 0.640 | 0.653 | 0.589 |
| c76 | (AC)_8_ | F:GTCATTTCATCCCTGGCTGT | R:ACCAAAGTTTCCTGACCCG | 239–267 | 14 | 2.558 | 119 | 0.679 | 0.698 | 0.655 |
| c77 | (AG)_8_ | F:ATCAGTCCTTTTTCGGCCTT | R:CACTCTCCCGGATCACATTT | 182–204 | 9 | 2.271 | 71 | 0.576 | 0.592 | 0.563 |
| c90 | (CT)_8_ | F:GCGAAGAAAACAAGTCTCGG | R:CTTGTTGCGTGGTCTTGAAA | 290–304 | 7 | 1.647 | 41 | 0.359 | 0.700 | 0.651 |
| c96 | (CT)_8_ | F:GGAGATTGTGGAGAAGCAGC | R:AAAAACCCTCCCAAACCATT | 206–220 | 8 | 2.085 | 46 | 0.539 | 0.570 | 0.509 |
| c97 | (GA)_8_ | F:ACCGTTTCATTAACCGCTCC | R:AGAAATCACGCCTCTCTCCA | 272–306 | 17 | 2.027 | 135 | 0.473 | 0.649 | 0.630 |
| c99 | (GTA)_7_ | F:CCCATGGCTTTGTCAGATTT | R:CCGCTTGTCCCTACACTCAT | 248–283 | 11 | 2.350 | 140 | 0.627 | 0.800 | 0.774 |
| c100 | (TGG)_6_ | F:TCCTTCTCCGCATCATCTCT | R:CACGAGTCATCACCAAATCG | 290–305 | 6 | 2.301 | 34 | 0.620 | 0.622 | 0.558 |
| c112 | (ATC)_6_ | F:CCAAAGGCCAAACTGTTGTT | R:TCTCAAGATGCTGCTTCCCT | 311–359 | 12 | 2.569 | 101 | 0.683 | 0.721 | 0.682 |
| c115 | (TTA)_7_ | F:TTGCTTCCTTCCTTCCTTGA | R:GGTTTGGCCTGGTTTTAGGT | 200–221 | 8 | 2.246 | 56 | 0.584 | 0.681 | 0.625 |

A, mean number of alleles per locus; Ai, mean number of different alleles per individual and locus; F, fixation index; G, mean number of four allele genotypes at a locus; He, expected heterozygosity; Ho, observed heterozygosity; N, sample size; PIC, polymorphism information content; SSR, simple sequence repeat.
