# Supplementary material for: Analysis of the genetic diversity and population structure of Salix psammophila based on phenotypic traits and simple sequence repeat markers
Source: PeerJ. 2019 Feb 18;7:e6419. doi: 10.7717/peerj.6419 (PMC6383557; doi:10.7717/peerj.6419)
Supplement: Supplemental Information 4 — BA, branch angle; GD, ground diameter; LA, leaf area; LL, leaf length; LP, leaf petiole; LPE, leaf perimeter; LW, leaf width; PH, plant height. Means with different letters within a column are significantly different according to Duncan test (P < 0.01). [file peerj-07-6419-s004.docx]

**Table S2.** Statistical parameters (mean ± standard deviation) of phenotypic traits in 17 *S. psammophila* populations. BA, branch angle; GD, ground diameter; LA, leaf area; LL, leaf length; LP, leaf petiole; LPE, leaf perimeter; LW, leaf width; PH, plant height. Means with different letters within a column are significantly different according to Duncan test (P<0.01).

| **Population** | **Trait** | | | | | | | | |
| --- | --- | --- | --- | --- | --- | --- | --- | --- | --- |
|  | **LL** | **LA** | **LPE** | **LW** | **LL/LW** | **LP** | **BA** | **PH** | **GD** |
| P1 | 5.45±0.97  EF | 1.65±0.49  EFG | 12.04±2.06  E | 0.37±0.07  BCDE | 14.92±3.12  E | 0.43±0.1  D | 37.62±4.31  A | 241.15±33.41AB | 14.2±5.32  ABCDE |
| P2 | 5.92±0.99  CDEF | 1.81±0.54  DEFG | 12.92±2.14  CDE | 0.4±0.07  ABC | 15.41±2.85  DE | 0.5±0.12  BCD | 37.33±4.22A | 232.86±27.64ABC | 13.60±4.97BCDEF |
| P3 | 7.25±2.25  A | 2.5±1.31  ABC | 15.6±4.8  AB | 0.41±0.1  AB | 17.6±2.91  BCDE | 0.64±0.24  B | 33.47±3.77BC | 217.66±42.1  BCD | 14.94±6.37  ABC |
| P4 | 6.82±1.67  ABC | 2.06±0.75  CDEF | 14.88±3.68  ABCD | 0.37±0.07  CDE | 19.29±5.19  BC | 0.6±0.24  BCD | 26.72±4.35F | 203.88±28.73D | 13.08±4.96  CDEF |
| P5 | 6.54±2.11  ABCD | 2.15±1.15  CDE | 14.12±4.63  ABCDE | 0.4±0.08  ABC | 16.76±4.45  CDE | 0.54±0.19  BCD | 26.89±4.99F | 212.21±35.58CD | 12.64±3.49  DEF |
| P6 | 5.61±1.17  DEF | 1.77±0.68  DEFG | 13.05±2.67  CDE | 0.32±0.08  FG | 18.1±3.54  BCDE | 0.51±0.13  BCD | 30.26±3.77DE | 217.21±31.58BCD | 12.12±3.66  EFGH |
| P7 | 5.47±1.12  EF | 1.59±0.47  FG | 12.68±2.54  DE | 0.31±0.06  G | 18.49±4.63  BCD | 0.5±0.12  BCD | 35.77±6.71AB | 221.84±39.75BCD | 12.39±3.2  EFG |
| P8 | 5.27±1.1  F | 1.49±0.43  G | 12.11±2.49  E | 0.3±0.06  G | 18.14±4.96  BCDE | 0.47±0.12  BCD | 26.74±2.47F | 220.28±33.51BCD | 11.97±3.39  FGH |
| P9 | 5.92±1.13  CDEF | 1.63±0.59  EFG | 13.45±2.56  BCDE | 0.26±0.06  H | 23.56±5.8  A | 0.53±0.13  BCD | 29.32±3.49DEF | 258.59±40.19A | 15.33±5.62  AB |
| P10 | 5.6±1.2  DEF | 1.56±0.52  FG | 12.84±2.99  CDE | 0.31±0.05  G | 18.66±4.56  BCD | 0.53±0.18  BCD | 35.42±5.39AB | 236.66±39.43ABC | 12.22±2.94  EFG |
| P11 | 6.16±0.82  BCDEF | 2.06±0.33  CDEF | 14.37±2.05  ABCDE | 0.39±0.03  BC | 16.15±2.7  CDE | 0.62±0.14  BC | 37.4±3.67  A | 208.48±36.71CD | 11.67±4.51  FGH |
| P12 | 5.94±1.25  BCDEF | 1.81±0.55  DEFG | 13.32±2.77  BCDE | 0.34±0.06  EFG | 18.66±5.98  BCD | 0.50±0.17  B | 28.87±3.3  DEF | 175.14±27.76E | 10.27±3.06  GHI |
| P13 | 6±0.94  BCDEF | 1.89±0.43  DEFG | 13.57±2.24  BCDE | 0.38±0.11  BCD | 17±3.28  BCDE | 0.51±0.22  BCD | 27.89±3.9  EF | 177.89±36.2  E | 8.55±1.97  I |
| P14 | 6.36±1.14  ABCDE | 2.1±0.67  CDEF | 13.41±2.98  BCDE | 0.35±0.08  DEF | 19.21±4.38  BC | 0.52±0.13  BCD | 29.7±4.44  DEF | 173.26±33.11E | 8.78±1.78  I |
| P15 | 6.96±1.39  AB | 2.69±0.78  AB | 15.29±2.9  ABC | 0.38±0.06  BCD | 18.81±4.07  BC | 0.54±0.15  BCD | 30.5±5.55  CDE | 176.66±20.48E | 9.99±2.57  HI |
| P16 | 7.17±1.31  A | 2.24±0.6  BCD | 16.4±5.38  A | 0.36±0.06  CDEF | 20.15±3.32  B | 0.44±0.11  CD | 28.11±2.31EF | 219±52.41  BCD | 14.62±4.88  ABCD |
| P17 | 7.33±0.63  A | 2.74±0.38  A | 16.54±1.52  A | 0.43±0.17  A | 18.7±2.7  BCD | 0.86±0.11  A | 31.57±5.11CD | 217.48±40.31BCD | 16.29±7.22  A |
| Mean ± Stdev | 6.23±1.46 | 1.99±0.77 | 13.95±3.41 | 0.36±0.09 | 18.29±4.53 | 0.54±0.18 | 31.29±5.75 | 212.2±42.74 | 12.50±4.78 |
