# Supplementary material for: Analysis of the genetic diversity and population structure of Salix psammophila based on phenotypic traits and simple sequence repeat markers
Source: PeerJ. 2019 Feb 18;7:e6419. doi: 10.7717/peerj.6419 (PMC6383557; doi:10.7717/peerj.6419)
Supplement: Supplemental Information 5 — The data are from general linear mixed models. numDF: numerator degrees of freedom. denDF: denominator degrees of freedom. Estimate, t- and P-values from the mixed model. [file peerj-07-6419-s005.docx]

**Table S3**. Reponses of phenotypic traits to sex of *S. psammophila.*

The data are from general linear mixed models. numDF: numerator degrees of freedom. denDF: denominator degrees of freedom. Estimate, t- and P-values from the mixed model.

| Trait | Sex | | | | |
| --- | --- | --- | --- | --- | --- |
|  | Estimate | numDF | denDF | *t* | *P* |
| LL | -0.0372 | 1 | 60.3308 | -0.2490 | 0.8040 |
| LA | 0.0213 | 1 | 82.7529 | 0.2840 | 0.7770 |
| LPE | -0.1559 | 1 | 89.3826 | -0.4800 | 0.6320 |
| LW | 0.0043 | 1 | 476.1000 | 0.5920 | 0.5540 |
| LL/LW | -0.5075 | 1 | 9.2451 | -1.0630 | 0.3150 |
| LP | -0.0135 | 1 | 479.6466 | -0.8000 | 0.4240 |
| BA | -0.5677 | 1 | 16.5200 | -1.0560 | 0.3060 |
| PH | -0.7026 | 1 | 15.9365 | -0.1640 | 0.8720 |
| GD | 0.4545 | 1 | 316.1013 | 0.8870 | 0.3750 |
