# Supplementary material for: Analysis of the genetic diversity and population structure of Salix psammophila based on phenotypic traits and simple sequence repeat markers
Source: PeerJ. 2019 Feb 18;7:e6419. doi: 10.7717/peerj.6419 (PMC6383557; doi:10.7717/peerj.6419)
Supplement: Supplemental Information 6 [file peerj-07-6419-s006.docx]

**Table S4**. Estimated breeding values and order for phenotypic traits of *S. psammophila.*

| Trait | Breeding value (Genets ID) | | | | |
| --- | --- | --- | --- | --- | --- |
|  | 1 | 2 | 3 | 4 | 5 |
| LL | 0.811(478) | 0.701(490) | 0.68(487) | 0.652(466) | 0.616(488) |
| LA | 0.439(478) | 0.423(490) | 0.389(473) | 0.389(466) | 0.383(487) |
| LPE | 1.876(478) | 1.642(490) | 1.559(487) | 1.526(466) | 1.435(481) |
| LW | 0.01(473) | 0.01(481) | 0.01(467) | 0.009(490) | 0.009(466) |
| LL/LW | 1.821(286) | 1.638(240) | 1.528(228) | 1.508(471) | 1.414(162) |
| BA | 5.809(59) | 5.289(19) | 5.186(23) | 5.11(57) | 4.993(14) |
| LP | 0.229(490) | 0.226(488) | 0.225(478) | 0.224(487) | 0.211(483) |
| PH | 10.87(11) | 10.189(2) | 9.696(29) | 9.569(28) | 9.526(246) |
| GD | 3.429(29) | 3.269(487) | 3.253(490) | 3.226(486) | 3.219(489) |
